# The Positive Impact of Joint Activities on Students Attitudes Toward Peers With Disabilities

**DOI:** 10.3389/fpsyg.2021.690546

**Published:** 2021-09-08

**Authors:** Ghaleb H. Alnahdi, Susanne Schwab, Ayman Elahdi, Aminah H. Alnahdi

**Affiliations:** ^1^Department of Special Education, College of Education, Prince Sattam Bin Abdulaziz University, Alkharj, Saudi Arabia; ^2^Center for Teacher Education, University of Vienna, Vienna, Austria; ^3^North-West University, Vanderbijlpark, South Africa; ^4^Ministry of Education, Cairo, Egypt; ^5^Department of Special Education, College of Education, King Saud University, Riyadh, Saudi Arabia

**Keywords:** attitudes, children with disabilities, SEN, inclusive education, Saudi Arabia, contact, CATCH

## Abstract

**Background:** Attitudes toward peers with disabilities play a crucial role in implementing inclusive education. This study examines how students' attitudes are associated with gender; having relatives with disabilities and the frequency of contact with them; attending a class that includes students with special educational needs (SEN); and having previous contact with SEN students through joint activities.

**Methods and Procedures:** The participants included 652 elementary school students (grades 4–6) who completed a short version of the Chedoke-McMaster Attitudes Toward Children with Handicaps (CATCH) questionnaire in Arabic.

**Outcomes and Results:** Students in inclusive classes express more positive attitudes in comparison with students in regular classes. However, previous contact through joint activities was associated with more positive attitudes. Females were more likely to hold positive views than males. Having relatives with disabilities had no effect; however, a high frequency of contact with them was associated with more positive perspectives.

**Conclusions and Implications:** The findings on students' attitudes indicate that joint activities between students with and without disabilities are important to promote positive attitudes. Planned opportunities to increase frequent contact, and to promote joint activities between students with and without SEN are recommended.

## Highlights

– Previous contact through joint activities is linked to more positive attitudes.
– Girls are more likely to hold positive attitudes than boys.
– Students in inclusive classes hold more positive attitudes than those in regular classes.
– Frequent of contact with relatives with disabilities associated with more positive attitudes.

## Introduction

Enrolling students with special educational needs (SEN) in regular classrooms is a shared goal in countries around the world (such as states in Europe, the US, Saudi Arabia, and South Africa). Diverse laws and policies push for reducing the proportion of SEN students in segregated school settings (e.g., special schools). The Salamanca Statement of 1994 (UNESCO, 1994) and the United Nations Convention on the Rights of Persons with Disabilities (UNCRPD, 2006) have become key documents that promote inclusive education globally. These documents are in line with research outcomes indicating that SEN students have more positive academic development in inclusive classes compared to SEN students in special schools (Ruijs and Peetsma, 2009; Oh-Young and Filler, 2015). According to a review by the European Agency for Special Needs Inclusive Education (2018), in addition to learning outcomes, social development and post-school options are higher for SEN students that attend inclusive classes compared to SEN students from special schools. Having SEN influences students' school lives immensely. Research has identified SEN students in inclusive classes as “at-risk students” in terms of social participation (Koster et al., 2009; Bossaert et al., 2013; Schwab, 2018).

### Attitudes Toward Inclusive Education

Even though political agendas may advocate for inclusion relatively clearly, applying it reveals certain challenges. It is important to identify aspects that affect the successful implementation of inclusive education in daily practice. The literature (Avramidis and Norwich, 2002; De Boer et al., 2011; Schwab et al., 2018) has emphasized the attitudes of the actors involved (e.g., teachers and students) as key factors. Therefore, it is not surprising that attitudes toward inclusive schooling have emerged as one of the top popular themes in recent research (De Vroey et al., 2016; Lüke and Grosche, 2018). Eagly and Chaiken (1998) ABC model of attitudes expresses various levels of acceptance that are linked to behaviors (for information on cognitive dissonance theory see Festinger, 1957; for details on the theory of planned behavior, see Ajzen and Fishbein, 1977). Their model contains three components: (1) *Affective*, which includes one's feelings about an object; (2) *Behavioral*, which refers to how one's attitude about the object impacts behavioral intentions; and (3) *Cognitive*, which involves one's belief about the object.

### Predictors of Students' Attitudes

Current research and policies indicate that it is important to give students the chance to express their feelings and views. Recent studies have started to take students' perspectives into account. Previous investigations show that the type of disability a child has influences peoples' perceptions of inclusive education. Students with socio-emotional disorders are often the targets of negative views, while attitudes toward students with learning disabilities are more positive. This effect has been demonstrated across different samples such as teachers, students, and parents (for an overview see Schwab et al., 2018). De Boer et al. (2012a) revealed the impact on students' views, which has been confirmed by several studies (e.g., Schwab, 2017; Hellmich and Loeper, 2018; Schwab et al., 2018). Moreover, most research has found a gender effect, implying that female students tend to hold more positive perceptions of inclusive schooling than males (for an overview see De Boer et al., 2012a and Schwab et al., 2018). In addition, contact with peers or others with disabilities is considered to predict attitudes. This is theoretically underpinned by the intergroup contact hypothesis (Allport, 1954; Pettigrew and Tropp, 2006), which assumes that contact reduces intergroup prejudice. For instance, students from inclusive classes (where students with and without disabilities are educated together) would have a more positive attitudes toward their peers with disabilities, as they have more contact with such students compared to those from regular classes. However, empirical evidence for this is lacking. While Nowicki and Sandieson (2002) verified that students from inclusive classes have a more positive outlook, Schwab (2015) did not find significant group differences; Gash et al. (2000) even discovered a paradox effect. Petry (2018) suggested that being educated together with students with autism spectrum disorder (ASD) is linked to a more negative perception of students with ASD compared to peers from classes with no intergroup contact in the class. Schwab (2017) implied that there is a positive effect of being educated together with SEN students, but only if their classmates are able to select them for joint activities (e.g., working together on school assignments). It was concluded that simply being placed in the same class does not improve attitudes; rather, there needs to be non-superficial contact based on free choice. Regarding the case of Saudi Arabia, Alnahdi (2019) demonstrated that contact with peers with disabilities at the school level had a positive impact on students' attitudes toward them. Abdulrahman (2018) found that having relatives with disabilities and the frequency of contact with them are associated with more positive perspectives among female high school students. Barakat (2014) and Al-Khwaldah (2015) found that female college students had more positive views than their male counterparts.

In general, several studies have demonstrated a positive connection between attitudes and contact with people with disabilities (e.g., relatives) (see MacMillan et al., 2014). Schwab et al. (2018) showed that the more contact students have with their peers with disabilities (both learning and socio-emotional disorders), the more positive their outlook. Hellmich and Loeper (2018) revealed similar effects.

Despite that many researchers in education and psychology tend to explore students' attitudes and the factors affecting them, Arabic-speaking countries have produced few studies in this area. This study thus examine the impact of gender, as well as having relatives with disabilities and the frequency of contact with them, on students' attitudes toward their peers with disabilities. This study will help to close this gap by portraying an under-represented sample in English-language research.

### Objectives

Our main objective is to investigate Saudi students' attitudes toward their peers with disabilities. With regard to predictors of attitudes, this study made the following assumptions based on prior literature:

1. Girls tend to hold a more positive attitude compared to boys.
2. Previous contact has a positive influence on students' attitudes.
  a. Students from inclusive classes hold more positive views than students from regular classes.
  b. Previous contact through joint activities is positively linked to students' perceptions.
  c. Students that have frequent contact with relatives with disabilities hold more positive attitudes than those with less frequent contact.

This study also examine psychometric qualities to answer the above questions, based on the attitude scale from the Chedoke-McMaster Attitudes Toward Children with Handicaps (CATCH) questionnaire.

## Methodology

### Sample

The sample in this study were culled from 29 classes at 9 elementary schools in the Riyadh region. In total, 652 students participated. Data were collected from both boys' and girls' schools, as the educational system in Saudi Arabia is separated by gender. The sample was comprised of 258 boys and 394 girls. The participants were from inclusive (*n* = 447, 283 girls, and 164 boys) and regular classes (*n* = 205, 111 girls, and 94 boys). Inclusive classes include at least one SEN student. All of the inclusive classes in this study have SEN students with learning disabilities who receive individualized support from special education teachers in separate resource rooms for a few hours during the week. Regular classes do not include SEN students. Before this study was conducted, an approval was received from the institutional review board (IRB) headed by the Deanship of Scientific Research at Prince Sattam Bin Abdulaziz University. Next, we obtained approval from school managers and parents' consent to administer the CATCH.

### Instruments

#### Students' Attitudes Toward Peers With Disabilities

A short version of the CATCH scale was employed to measure children's attitudes toward their peers with disabilities (Rosenbaum et al., 1986). This scale and adapted versions of it have been used with different samples from various countries (for the Netherlands, see De Boer et al., 2012b; for Belgium, see Bossaert and Petry, 2013; for Austria, see Schwab et al., 2018; for Germany, see Hellmich and Loeper, 2018;). Since the original scale is relatively long (36 items in total), researchers would rather use shorter versions of the CATCH. For the present study, we used a shorter form developed by Schwab (2018). This short version was limited to four items (e.g., *I would be happy to have [name of the person] for a friend*) and only included the affective and behavioral components of attitude. The version proposed by Schwab et al. (2018) uses case descriptions of different types of disabilities: intellectual, learning, physical, and social/emotional. These case descriptions were developed by Anke de Boer and approximate the previous work of De Boer et al. (2012b). For example:

*Markus is a boy your age and has just arrived in town. He goes to the same school as you. Markus has just started reading and writing, but has a lot of difficulty with math. He can run and play like other children, but sometimes forgets the rules of the game. He needs much more time to solve problems and complete activities than the other children, and sometimes forgets things. At times, it is hard to understand what Markus says. For part of the day, Markus receives learning support outside the classroom*.

Schwab et al. (2018) obtained satisfactory high reliability scores and strong factor loadings on a single factor for this short version for both students with and without SEN in all different case descriptions. This study has used the same items from the Arabic version of CATCH after the case description (see Alnahdi, 2020; Alnahdi et al., 2020). For the case descriptions, we translated the children's names into Arabic ones (e.g., Saeed instead of Markus). Two different versions were used: a female name for the girls' sample and a male name for the boys' sample. In both the boys' and girls' questionnaire, four types of disabilities were investigated: intellectual, learning, physical, and social/emotional. We covered all four in both types of classes and across gender to minimize any effect they may have. The translation process was based on the steps recommended by Beaton et al. (2000). First, two bilingual researchers in the field of special education carried out an English to Arabic translation. Then, a third bilingual expert with a specialty in English translated the Arabic version to English. Two of the researchers compared the new English version with the original English version, and made minimal changes to the Arabic version. Next, a pilot study of the scale was conducted with 53 students to make sure that it was clearly understood. The Cronbach's alpha for the pilot study was 0.743.

#### Contact With Relatives With Disabilities

The frequency of contact for students who have relatives with disabilities was categorized in two groups: high (5 times or more per year, *N* = 138) and low (5 times or less per year, *N* = 83).

#### Contact Through Joint Activities

The variable of having previous contact through joint activities was measured by this item: *I have often worked on school projects with someone like Saeed*. This single item has been used by other researchers (Hellmich and Loeper, 2018; Schwab et al., 2018). The students responded to this item on a 4-point Likert scale.

### Data Analysis

Different statistical analyses were used in this study to examine this study assumptions. The reliability was examined by calculating alpha Cronbach. The means and standard deviations were calculation by different independent variables (see Table 1). Multiple regression models were conducted to examine the predictability of different independent variables on students' attitudes in this study. The IBM SPSS package software used to analysis this study data.

**Table 1:** Descriptive statistics by independent variables.

		**M (SD)**	**N**
Gender	Girl	3.49 (0.76)	394
	Boy	3.03 (1.04)	258
	Inclusive	3.38 (0.85)	447
Classroom	Regular	3.15 (1)	205
Relative with disability	Yes	3.31 (0.90)	221
	No	3.30 (0.91)	430
Overall		3.31 (0.91)	652

## Results

### Reliability and Validity of the Catch

The Cronbach's alpha reliability was 0.894 for the scale, indicating good internal consistency of the scale (George and Mallery, 2003). In addition, a confirmatory factor analysis (CFA) was conducted to examine the scale's construct validity. Good fit indices were obtained using the non-significant chi-square test. The comparative fit index (CFI) was >0.95, the root mean square error of approximation (RMSEA) was <0.8, and the adjusted goodness of fit index (AGFI) was >0.90 (Schermelleh-Engel et al., 2003; Byrne, 2010). The chi-square was not significant; $\chi_{\left(1,\text{N}=652\right)}^{2}$ = 3.298; *p* = 0.069; RMSEA = 0. 059; CFI = 0.999; and AGFI = 0.975. In sum, the Arabic short version of CATCH showed good psychometric properties, to be used to measure students' attitudes toward their peers with disabilities.

Table 1 showed that the overall mean score for the CATCH in the sample was M = 3.31 and SD = 0.91. To calculate the percentages of students' views among the three categories (positive, neutral, and negative attitudes), we used a 4-point Likert scale; the midpoint is 2.5. The range is 3. Through dividing it by three categories, a mean score of 2.0 or lower was considered negative; a mean score of 3.0 or higher was considered positive, and a mean higher than 2 and < 3 was considered neutral. Around 75% of students fell within the range of positive attitudes; around 13% had a negative perception, and 12% had neutral views.

### Predictors of Students' Attitudes

A hierarchical multiple regression analysis was used to examine predictors of students' attitudes. The analysis was conducted in three steps. In the first model, we wanted to explore inclusive classes only, without controlling for contact through joint activities. We added contact in joint activities to the second model. In the third model, we limited the sample to those that have relatives with disabilities to check for the impact of frequency of contact with them.

In the first model, three independent variables were entered as predictors: (1) class setting (inclusive classes versus regular classes); (2) relatives with disabilities; and (3) gender. We found a significant regression model (*F*_(3,645)_ = 18.775, *p* < 0.01), with an R^2^ of 0.08. This means that this model, with the three variables, explained around 8% of the variation in students' perspectives. By checking the contribution of each predictor in this model, it was found that both class setting and gender were significant predictors, while having relatives with disabilities did not significantly contribute to predicting attitudes (see Table 2). Students from inclusive classes expressed more positive attitudes (M = 3.38, SD = 0.85) compared to those in regular classes (M = 3.15, SD = 1). Girls had more positive attitudes (M = 3.49, SD = 0.76) than boys (M = 3.09, SD = 1.04).

**Table 2:** Multiple regression models to predict students' attitudes (CATCH) *n* = 652.

**Model**	**Predictor**	**Unstandardized Coefficients**		**Standardized Coefficients**	**t**	**Sig**.	Partial R^*^
		**B**	**Std. Error**	**Beta**			
1	(Constant)	2.863	0.198		14.479	0.000	
	Type of class	−0.202	0.073	−0.105	−2.765	0.006	−0.108
	Gender	0.465	0.069	0.254	6.707	0.000	**0.255**
	Relative with disability	−0.038	0.071	−0.020	−0.537	0.591	−0.021
2	(Constant)	1.301	0.176		7.407	0.000	
	Type of class	0.055	0.059	0.029	0.933	0.351	0.037
	Gender	0.360	0.055	0.197	6.522	0.000	**0.249**
	Relative with disability	0.019	0.057	0.010	0.344	0.731	0.014
	Contact through joint activities	0.455	0.023	0.605	19.595	0.000	**0.611**

* *Partial R = correlation between the predictor with CATCH after controlling for all other variables in the model, Bold = significant predictor at p < 0.01, Type of class = inclusive or regular*.

In the second model, we added the variable of whether students had previous contact with SEN students through joint activities. The model improved significantly by an increase in the R^2^ of 0.420. This means that this model would explain 42% of variations in attitudes, more than in the first model. After adding contact through joint activities to the model, the inclusive classroom variable was no longer significant. Two significant variables were in the second model: previous contact through joint activities and gender. Students with more previous contact in joint activities had a significant partial correlation of R = 0.611. This high correlation between students' attitudes and previous contact through joint activities occurred after controlling for the variables in the model. Which means that joint activities variable explained around 37% unique variation of the explained variance in attitudes after controlling of other variables in the model.

### Relatives With Disabilities

An additional step was conducted to examine why having relatives with disabilities did not explain significant variations in students' attitudes. A third model was performed by adding frequency of contact for those that have relatives with disabilities (Table 3). We did not add it to the second model because it would limit the sample to those that have relatives with disabilities. Thus, the third model is limited to participants that have relatives with disabilities. The model improved significantly by an increase in R^2^ of 0.020. By adding the frequency of contact variable, the model explained around 2% more of variations in attitudes.

**Table 3:** Multiple regression models to predict attitudes of students with relatives with disabilities *n* = 221.

**Model**	**Predictor**	**Unstandardized Coefficients**		**Standardized Coefficients**	**t**	**Sig**.	Partial R^*^
		**B**	**Std. Error**	**Beta**			
1	(Constant)	2.966	0.428		6.937	0.000	
	Type of class	0.024	0.119	0.013	0.200	0.842	0.013
	Gender	0.378	0.115	0.217	3.286	0.001	**0.216**
	Relative with disability	−0.304	0.349	−0.057	−0.871	0.385	−0.058
2	(Constant)	1.247	0.351		3.55	0.000	
	Type of class	0.108	0.091	0.060	1.191	0.235	0.080
	Gender	0.305	0.088	0.174	3.481	0.001	**0.228**
	Relative with disability	0.000	0.266	0.000	0.000	1.000	0.000
	Contact through joint activities	0.486	0.038	0.643	12.80	0.000	**0.654**
3	(Constant)	0.720	0.389		1.851	0.066	
	Type of class	0.106	0.089	0.058	1.187	0.237	0.080
	Gender	0.352	0.088	0.201	4.019	0.000	**0.262**
	Relative with disability	0.101	0.264	0.019	0.383	0.702	0.026
	Contact in joint activity	0.481	0.037	0.636	12.85	0.000	**0.656**
	Frequency of contact	0.253	0.087	0.147	2.923	0.004	**0.194**

* *Partial R = correlation between the predictor with CATCH after controlling for all other variables in the model, Bold = significant predictor at p < 0.01, Type of class = inclusive or regular*.

In sum, the three variables significantly predicted students' views of their peers with disabilities: (1) previous contact through joint activities; (2) gender; and (3) frequency of contact with relatives with disabilities.

## Discussion

It was important to carry out this study given that governmental decisions, pushed forward toward inclusive schooling, need to be supported by those directly affected by school reforms. Previous literature mainly explored teachers' attitudes toward inclusive schooling and did not consider students' views. Since students' perceptions of their peers with disabilities seem to have a high impact on the latter's daily lives (e.g., with regard to social inclusion, see Schwab, 2018), it is vital to identify predictors of students' attitudes.

In general, our outcomes indicate that Saudi students tend to hold rather positive attitudes toward peers with disabilities. The percentage of students with a negative perception was quite low. This finding would need to be confirmed in future studies using a longitudinal design to identify causal directions. Moreover, future research could investigate if a more positive attitude not only leads to a higher intention of having contact, but also to more contact in real-life situations. This would underscore the importance of students' attitudes toward peers with disabilities. In the line with this finding, it was found that students in inclusive classes hold more positive attitudes in comparison to students in and regular classes. Even if the class setting (regular versus inclusive) predicted students' attitudes, the effect was quite small. Moreover, after adding contact through joint activities as a predictor, it showed a significant impact on students' attitudes. This implies that it may have been significant in the first model because students in inclusive classes have more chances to come into contact with their SEN peers through joint activities than students in regular classes, not simply because they may be in in the same classes as SEN students. This confirms the findings of Schwab (2017), which indicate that students need to have in-depth contact. Inclusive classes have huge potential to increase the chances of joint activities between students with and without disabilities. Next to contact with peers in class, contact with relatives with disabilities can positively influence students' attitudes. However, a similar pattern has been shown for having relatives with disabilities. In general, having relatives with disabilities did not affect students' perceptions. However, a high frequency of contact with them was associated with more positive views. Hence, contact in one's spare time (including the quantity and quality of the contact) may play a major role.

In line with several prior studies, in our sample, girls showed a more positive view compared to boys. This is consistent with Schwab (2018) results, where girls had a more positive attitude toward peers with disabilities. In addition, other studies have found that females of different ages tend to have more positive perceptions of their peers with disabilities (Rosenbaum et al., 1988; Vignes et al., 2009; De Boer et al., 2012a; Barakat, 2014; Alnahdi et al., 2019).

## Implications and Limitations

Several implications can be drawn from the literature review and from this study findings. First, school officials need to encourage teachers to plan and facilitate joint activities. This will increase the chances of influencing students' attitudes in positive way, especially when it comes to activities that are fun and competitive, in turn leading to a higher possibility of being welcoming toward SEN students. Second, the Ministry of Education has a role to play by enacting laws in support of special education teachers that allow them to conduct joint activities among students with and without various disabilities. Without such clear and explicit support, joint activities will remain mere individual efforts by teachers and will not be sustainable. Third, schools and the media must raise awareness in order to decrease stereotypes of those with disabilities, which normally affect what people think about minority groups (Rosenbaum, 2010). Fourth, continuing to increase the number of schools and classes where SEN students are integrated is critical as an initial opportunity to enhance chances for participation in joint activities. Without doing so, the options for joint activities will be limited. Fifth, future studies should explore the relationship between students' attitudes and their intentions to engage in joint activities both within and outside school. In addition, studying samples from different Arabic-speaking regions will help researchers understand students' views and the factors affecting them.

This study has a few limitations as well. The sample were collected from the Riyadh region, which might not represent student populations across Saudi Arabia. However, we made efforts to cover different regions in Riyadh, as it is the largest and most populated city in Saudi Arabia. Second, although the number of participants in this study is considered good, it may not be representative of students from various sub-groups. However, there is a strong representation of the three independent variables that were addressed in the statistical analysis.

## Data Availability Statement

The datasets generated for this study are available on request to the corresponding authors.

## Ethics Statement

The studies involving human participants were reviewed and approved by Prince Sattam bin Abdulaziz University. Written informed consent to participate in this study was provided by the participants' legal guardian/next of kin.

## Author Contributions

All authors listed have made a substantial, direct and intellectual contribution to the work, and approved it for publication.

## Conflict of Interest

The authors declare that the research was conducted in the absence of any commercial or financial relationships that could be construed as a potential conflict of interest.

## Publisher's Note

All claims expressed in this article are solely those of the authors and do not necessarily represent those of their affiliated organizations, or those of the publisher, the editors and the reviewers. Any product that may be evaluated in this article, or claim that may be made by its manufacturer, is not guaranteed or endorsed by the publisher.
